# Laparoscopic repair of parahiatal hernia after esophagectomy: a case report

**DOI:** 10.1186/s40792-017-0367-2

**Published:** 2017-08-23

**Authors:** Yuji Akiyama, Takeshi Iwaya, Fumitaka Endo, Takehiro Chiba, Takeshi Takahara, Koki Otsuka, Hiroyuki Nitta, Keisuke Koeda, Masaru Mizuno, Yusuke Kimura, Akira Sasaki

**Affiliations:** 1 Department of Surgery, Iwate Medical University School of Medicine, Iwate, Japan; 2 Department of Palliative Medicine, Iwate Medical University School of Medicine, Iwate, Japan

**Keywords:** Parahiatal hernia, Esophagectomy, Laparoscopic repair, Mesh repair

## Abstract

**Background:**

Diaphragmatic hernia is a potential complication of esophagectomy, which usually occurs as a hiatal hernia and more frequently after minimally invasive esophagectomy. Parahiatal hernia is a rare form of diaphragmatic hernia, and to the best of our knowledge, parahiatal hernia after esophagectomy has not been previously reported. Here, we report a case of parahiatal hernia after esophagectomy that was successfully managed laparoscopically.

**Case presentation:**

A 73-year-old man underwent thoracoscopic esophagectomy for esophageal cancer with gastric tube reconstruction via the posterior mediastinum. Postoperative morbidity was ileus, which required conservative treatment, and intestinal obstruction for which operation with laparotomy was necessary. He was admitted with abdominal pain and vomiting at 15 months after esophagectomy. Abdominal X-ray revealed colon gas in the intrathoracic space. A barium enema examination showed a transverse colon incarcerated in the intrathoracic space. The patient was preoperatively diagnosed with hiatal hernia after esophagectomy, and laparoscopic hernia repair was performed. During the surgery, the hiatus was found to be intact, and the defect was clearly separated from the left crus of the diaphragm. Parahiatal hernia was the operative diagnosis. The incarcerated colon was repositioned in the abdominal cavity, and the defect was repaired using a composite mesh.

**Conclusions:**

Laparoscopic surgery was found to be effective for the diagnosis and repair of parahiatal hernia.

## Background

Diaphragmatic hernia is a potential complication of esophagectomy, which usually occurs as a hiatal hernia and more frequently after minimally invasive esophagectomy. [1–4]. Parahiatal hernia is a rare form of diaphragmatic hernia [5], and parahiatal hernia after esophagectomy has not been previously reported. Here, we report a case of parahiatal hernia after esophagectomy that was successfully managed laparoscopically.

## Case presentation

A 73-year-old man was admitted to our hospital with a diagnosis of esophageal squamous cell carcinoma in the middle thoracic esophagus in October 2014. The clinical diagnosis was T4b (left main bronchus) N2M0 stage IIIC carcinoma, according to the seventh edition of the Union for International Cancer Control TNM Classification of Malignant Tumors. Accordingly, he was initially treated by triple induction chemotherapy comprising docetaxel, cisplatin, and 5-fluorouracil. Febrile neutropenia and neutropenic enterocolitis were observed as adverse events. Downstaging to T3N2M0 stage IIIB carcinoma was achieved after two courses of chemotherapy. Thoracoscopic esophagectomy for esophageal cancer with gastric tube reconstruction via the posterior mediastinum was performed in January 2015. An abdominal approach was applied using open laparotomy. A jejunostomy catheter was placed for early postoperative enteral nutrition. Ileus due to *Clostridium difficile* enteritis was a postoperative morbidity, with hepatic portal venous gas and sepsis on postoperative day (POD) 10. Left pleural effusion was pooled and treated with drainage between POD 11 and 25. The patient recovered and was discharged on POD 28. He was then identified as having intestinal obstruction with volvulus at the site of the removed jejunostomy catheter and underwent surgery with laparotomy to correct this in August 2015.

**Fig. 1:**
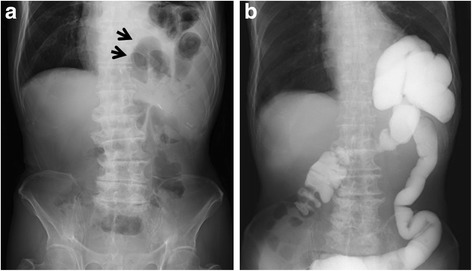
Abdominal X-ray and barium study. **a** Abdominal X-ray revealed colon gas in the intrathoracic space. **b** Barium enema examination revealed that the transverse colon was incarcerated in the intrathoracic space

**Fig. 2:**
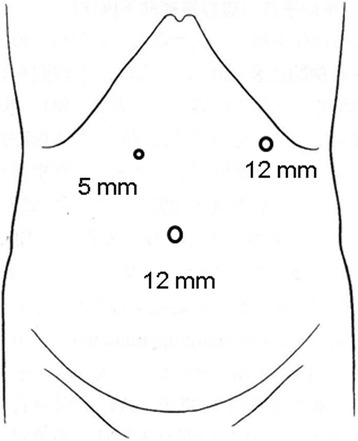
Port setting for hernia repair. In total, three ports were placed in the abdomen

**Fig. 3:**
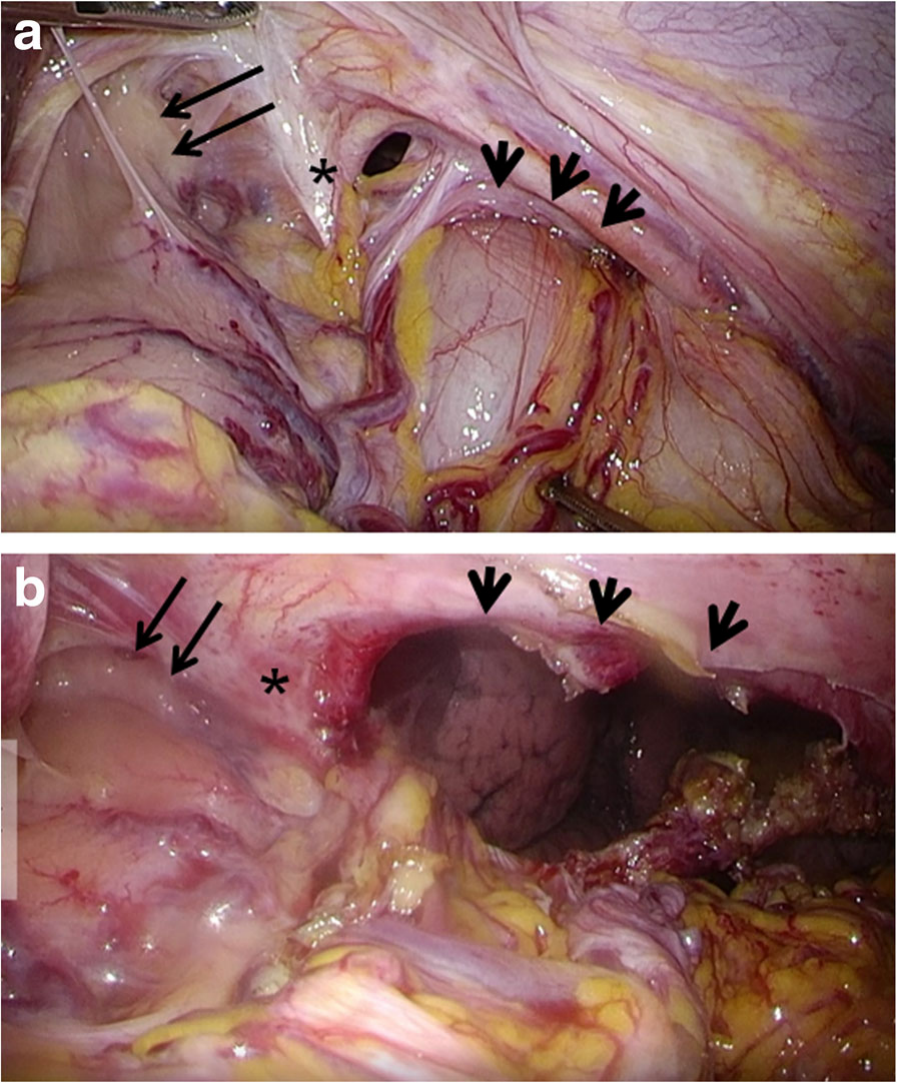
Intraoperative findings of parahiatal hernia. The defect (arrowheads) was clearly separated from the musculature of the left crus of the diaphragm (asterisks). The conduit of the gastric tube is shown by arrows. **a** A transverse colon was incarcerated in the hernia defect. **b** An 8 × 5 cm diaphragmatic defect was observed left lateral to the hiatus

**Fig. 4:**
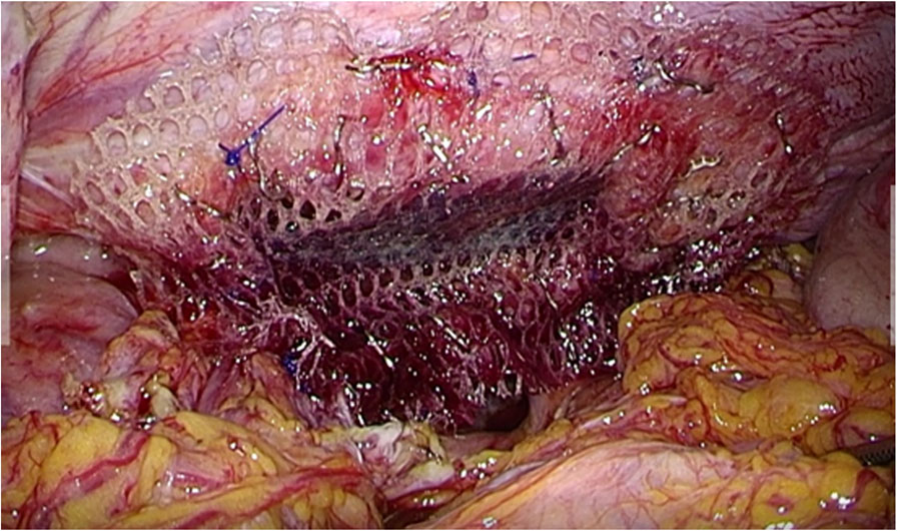
The defect was repaired using a composite mesh, fixed by sutures and a titanium hernia stapler

Subsequently, the patient was admitted to our hospital having presented with abdominal pain and vomiting in April 2016 (15 months after the esophagectomy). The abdominal X-ray revealed colon gas in the intrathoracic space (Fig. 1a). A barium enema examination showed the transverse colon to be incarcerated in the intrathoracic space (Fig. 1b). This finding was supported on chest and abdominal computed tomography (CT). The patient was diagnosed with hiatal hernia after esophagectomy and underwent laparoscopic hernia repair. Three ports were placed in the abdomen (Fig. 2). An 8 × 5 cm diaphragmatic defect was observed to the left of the hiatus laterally (Fig. 3). The left crus of the diaphragm was intact, and the defect was observed to be clearly separated from the left crus. Parahiatal hernia was the operative diagnosis. The incarcerated colon was repositioned in the abdominal cavity, and the defect was repaired using a composite mesh (Medtronic, Dublin, Ireland). The composite mesh was fixed along the circumference with a non-absorbable 2-0 polypropylene suture and titanium hernia stapler (Fig. 4). The operative time was 157 min, with 92 ml of blood loss. Although drainage was required for left pleural effusion after surgery, this was successfully resolved and the patient was discharged on POD 19. No recurrence of the hernia was observed 12 months after surgery.

### Discussion

There are two common forms of esophageal hiatal hernia: “sliding hiatal hernia” and “paraesophageal hernia” [5]. Another rare form of hiatal hernia is “parahiatal hernia,” which is characterized by the presence of a separate extrahiatal diaphragmatic defect with intervening normal crural muscle. Parahiatal hernia is a rare form of diaphragmatic hernia, and its exact incidence is unknown. Scheidler et al. reported that the incidence of parahiatal hernia was 0.2% in their study on patients undergoing surgery for hiatal hernia repair [5]. Palanivelu et al. also reported that four primary parahiatal hernias (0.35%) were identified in their case series involving 1127 patients with hiatal hernias who underwent fundoplication [6].

It is difficult to diagnose a parahiatal hernia preoperatively. It is difficult to make a distinction clinically between parahiatal and hiatal hernia, and the former is usually diagnosed by intraoperative findings. Scheidler et al. indicated that specific roentgenographic findings helped to distinguish between parahiatal and hiatal hernias, i.e., the position of the intrathoracic herniated abdominal viscus was moved noticeably to the left of the midline [5]. Though we observed this situation in our case, it was difficult to make an accurate diagnosis based solely on this finding. If the crural musculature between the hiatus and hernia orifice could be identified on an abdominal CT, it might aid in the diagnosis of parahiatal hernia. However, we could not confirm that the left crus and orifice were separated on CT. Thus, we could not make this distinction even when it was examined in detail retrospectively.

Usually, diaphragmatic hernia after esophagectomy occurs as a hiatal hernia [1]. We performed a literature search in PubMed using “parahiatal hernia” as the keyword. Only 20 adult cases have been documented. We did not find reports of secondary hernia after esophagectomy within a retrieval range. Choi et al. reported a case of “parahiatal hernia” following Ivor Lewis esophagectomy. The findings were not of parahiatal hernia but of hiatal hernia, with herniation of the colon through the hiatus [7]. Details of characteristics and treatment of 18 patients that we found from 1990 to 2016 on PubMed using the same keywords, including our case, are summarized in Table 1. We also diagnosed hiatal hernia after esophagectomy preoperatively in our patient. Enlargement of the hiatus during esophagectomy is a predisposing factor for hiatal hernia after esophagectomy [1]. Although we enlarged the hiatus slightly to insert a conduit and allow posterior mediastinum reconstruction during esophagectomy, the hiatus of the diaphragm was firmly and properly fixed during surgery for hernia repair in this case. A secondary parahiatal hernia may occur after traumatic injury to the diaphragm or after iatrogenic injury following previous surgery in the left upper quadrant of the abdomen [5]. As summarized in Table 1, there were five patients with secondary hernia in the previous reports, of whom three patients previously underwent fundoplication, one underwent transhiatal surgery, and one underwent treatment for left malignant pleural mesothelioma. Actually, almost patients were treated around the hiatus in previous surgeries. In our case, the patient had undergone abdominal surgery twice previously. Usually, we perform gastric mobilization and abdominal lymphadenectomy by hand-assisted laparoscopic surgery, followed by conduit formation extracorporeally through a small laparotomic incision. In this case, we could accomplish mobilization of the stomach and abdominal lymphadenectomy via the small incision. In this procedure, we divided the gastrosplenic ligament including the short gastric arteries with a vessel-sealing device. The diaphragm was possibly unknowingly injured by the tip of an energy device around the upper pole of the spleen during the operation. Therefore, we diagnosed secondary hernia in this case. Subsequently, the patient underwent surgery to resolve the intestinal obstruction via the same upper abdominal small incision. We assumed that the diaphragm had probably not been damaged during the surgical procedure in this operation because the surgical field was around the site of the removed jejunostomy catheter in the left lateral abdomen. The patient experienced ileus with pneumatosis intestinalis and hepatic portal venous gas owing to *C. difficile* enteritis after esophagectomy. The weight of the patient was 46 kg upon the first admission to our hospital with a 7-kg decrease during the previous 3 months. Furthermore, the patient showed a weight loss to 40 kg during neoadjuvant chemotherapy preoperatively. Although the patient did not show a drastic weight loss postoperatively, it was thought that he was under prolonged malnutrition, which might have made the tissue fragile. In addition, we thought his ileus might be related to parahiatal hernia formation. The patient underwent emergency surgery on the same day of the onset of bowel obstruction 7 months after esophagectomy. He suffered ileus with pneumatosis intestinalis and hepatic portal venous gas after esophagectomy, and he was not able to ingest a meal for a while. Continuous abdominal pressure due to ileus and subileus might gradually have extended the injured part of the diaphragmatic musculature and might have induced the parahiatal hernia.

**Table 1:** Patient characteristics and treatment for parahiatal hernia

Author	Case	Gender	Age	Symptom	Etiology: primary/secondary (previous surgery)	Defect size (cm)	Treatment	Postoperative course
Demmy et al. (1994) [12]	1	F	48	Upper abdominal pain	Primary	2	Left thoracotomy Primary closure	Discharge on POD 42
Rodefeld et al. (1998) [13]	2	F	64	Heartburn and regurgitation	Primary	5	Laparoscopic repair Primary closure Fundoplication	Discharge on POD 3 Asymptomatic on 15 months postoperatively
Scheidler et al. (2002) [5]	3	F	68	Postprandial nausea, emesis, and epigastric pain	Primary	ND	Laparoscopic repair Primary closure Fundoplication	Discharge on POD 2 Asymptomatic on 12 months postoperatively
	4	M	57	Postprandial, substernal chest pain	Primary	ND	Laparoscopic repair Primary closure Fundoplication	Postoperative course was the same as case 3 Asymptomatic on 4 years postoperatively
Palanivelu et al. (2008) [6]	5	M	32	Epigastric pain (50%), nausea (15%), vomiting (10%), heartburn (80%), postprandial bloating (25%)	Primary	8	Laparoscopic repair Mesh repair	Mean hospital stay was 5 days (2–8 days) Patients resumed their regular work schedule 10–14 days postoperatively
	6	M	55		Primary	18	Laparoscopic repair Mesh repair	
	7	M	29		Primary	30	Laparoscopic repair Mesh repair Fundoplication	
	8	M	65		Primary	16	Laparoscopic repair Mesh repair Fundoplication	
	9	F	45		Secondary (LF for GERD)	6	Laparoscopic repair Primary closure	
	10	M	70		Secondary (LTE for esophageal leiomyoma)	9	Laparoscopic repair Mesh repair	
	11	F	56		Secondary (LF for GERD)	6	Laparoscopic repair Primary closure	
	12	F	37		Secondary (LF for GERD)	8	Laparoscopic repair Mesh repair	
Ohtsuka et al. (2012) [14]	13	M	39	Epigastric pain, nausea, and vomiting	Primary	5	Laparoscopic repair Primary closure	ND
Takemura et al. (2013) [10]	14	M	70	Epigastric pain	Secondary (biopsy of pleura for mesothelioma)	3	Laparoscopic repair Primary closure	Discharge on POD 29
Lew et al. (2013) [11]	15	F	51	Epigastric pain and vomiting	Primary	3	Laparoscopic repair Mesh repair	Discharge on POD 5 Asymptomatic on 7 months postoperatively
Staerkle et al. (2016) [9]	16	M	71	Chest pain	Primary	ND	Laparoscopic repair Mesh repair Fundoplication	Discharge on POD 3 Mild symptomatic reflux on 2 years postoperatively
Koh et al. (2016) [8]	17	F	40	Epigastric pain	ND	5	Laparoscopic repair Primary closure	Discharge on POD 2
	18	F	51	Epigastric pain	ND	3	Laparoscopic repair Mesh repair Fundoplication	Discharge on POD 5
Our case	19	M	73	Abdominal pain	Secondary (reconstruction of gastric conduit)	8	Laparoscopic repair Mesh repair	Discharge on POD 19 Asymptomatic on 12 months postoperatively

*M* male, *F* female, *POD* postoperative day, *ND* not described, *LF* laparoscopic fundoplication, *GERD* gastroesophageal reflux disease, *LTE* laparoscopic transhiatal enucleation

In recent reports, almost all cases of parahiatal hernia were treated by laparoscopic repair of the hernia (Table 1) [6, 8–14]. The reason for this might be the fact that hiatal hernia was the preoperative diagnosis. In our case, laparoscopic surgery was effective because it allowed us to diagnose and repair a large diaphragmatic defect owing to sufficient working space and an increased field of view. Although the hernial defect was too large for direct suture, tension-free repair was achieved using a mesh.

## Conclusions

This was a rare case of parahiatal hernia following thoracic esophagectomy involving gastric tube construction via the posterior mediastinum. Laparoscopic surgery was found to be effective for the diagnosis and repair of a parahiatal hernia.

## Abbreviations

CT: Computed tomography
POD: Postoperative day
